# Comparing cancer stage at diagnosis between migrants and non-migrants: a meta-analysis

**DOI:** 10.1038/s41416-024-02896-0

**Published:** 2024-11-12

**Authors:** Adam Harvey-Sullivan, Sana Ali, Parveen Dhesi, Joseph Hart, Helena Painter, Fiona M. Walter, Garth Funston, Dominik Zenner

**Affiliations:** 1 Wolfson Institute of Population Health, Queen Mary University of London, London, UK; 2 Primary Care & Population Health Department, University College London, London, UK

**Keywords:** Neoplasm staging, Cancer epidemiology

## Abstract

**Background:**

Migrants face barriers accessing healthcare, risking delays in cancer diagnosis. Diagnostic delays result in later stage diagnosis which is associated with poorer cancer survival. This review aims to compare the differences in cancer stage at diagnosis between migrants and non-migrants.

**Methods:**

We conducted a systematic review and meta-analysis of three databases from 2000 to 2023 for studies conducted in OECD countries that compared stage at diagnosis between migrants and non-migrants. Meta-analysis compared odds ratios (OR) for early (stage I and II) stage at diagnosis. The *Risk of Bias in Non-randomised Studies of Exposure* tool was used to assess study quality.

**Results:**

41 of the 11,549 studies identified were included; 34 studies had suitable data for meta-analysis. Overall, migrants were significantly less likely to be diagnosed with early stage cancer compared with non-migrants (OR 0.84; 95% CI 0.78–0.91). This difference was maintained across cancer types, although only statistically significant for breast (OR 0.78; 95% CI 0.70–0.87) and prostate cancer (OR 0.92; 95% CI 0.85–0.99).

**Discussion:**

Published studies indicate that migrants are less likely to be diagnosed with early stage cancer. Variation by cancer type, study location and region of origin highlights the need for further research to understand these differences.

## Introduction

Worldwide, there are an estimated 280 million migrants, people who live in a country different from their country of birth. Migrants are heterogeneous coming from diverse backgrounds with a variety of reasons for their migration. Migrants can experience a range of specific problems and inequalities because of the circumstances of departure and arrival, including their cultural-linguistic and demographic specificities, their socioeconomic circumstances and their legal status [1, 2]. These issues can lead to or include barriers to accessing healthcare. In combination, these factors may lead to delays in the provision of timely healthcare including cancer diagnosis. Cancer diagnosis is a complex, multi-step process that is determined by patient, clinician, and healthcare system factors [3–5]. Any barriers to this complex process can delay diagnosis. Stage at diagnosis is a key factor in determining cancer survival [6]. A one-month delay in cancer treatment is associated with an estimated 10% increase in mortality [7].

Barriers to healthcare access for migrant populations may occur across multiple levels including impediments to entitlement, accessibility, and healthcare system responsiveness. Entitlement barriers refer to administrative and policy hurdles that obstruct healthcare coverage such as healthcare charging and a lack of awareness of eligibility criteria [8, 9]. For example, asylum seekers and refugees in Germany are only entitled to more than emergency care if they have been in the country for more than 15 months. Access barriers include inappropriate refusal of care and discrimination by staff [10, 11] as well as fear amongst migrants that accessing care may have legal repercussions including impacting their immigration status [12]. A 2023 survey in the USA found that 27% of undocumented immigrants and 8% of legal immigrants avoided accessing healthcare due to immigration-related fears [13]. Healthcare system responsiveness barriers include lack of interpreting services to bridge linguistic differences [14, 15]. A review of linguistically diverse migrants with cancer in Australia found pervasive and significant communication problems across the cancer care continuum that impaired the patients’ capacity to navigate the system and the clinicians’ capacity to provide adequate care [16].

There is a growing body of literature highlighting barriers to healthcare access for migrants, but how this applies to cancer diagnosis is poorly understood. Whilst challenges navigating the health system such as health literacy and communication difficulties may echo wider barriers to healthcare [16, 17], there may also be factors specific to the cancer diagnostic pathway. For example, around the world, beliefs and stigma around cancer vary as does the awareness of cancer-related symptoms and the importance of prevention and early detection [18]. Internationally, research on inequalities for migrants has focused on cancer screening rather than symptomatic diagnosis [19, 20]. Existing literature exploring delays in cancer diagnosis for marginalised groups mainly focuses on ethnicity rather than migration status [21–24]. However, there are factors distinct to migration including typology and immigration status that may risk delaying cancer diagnosis. Therefore, there is a need to investigate the symptomatic cancer diagnostic pathway for migrants.

The aim of this study is to conduct a systematic review and meta-analysis comparing cancer stage at diagnosis between migrants and non-migrants. We hypothesise that migrants are less likely to have early stage cancer at diagnosis than non-migrants. This hypothesis is based on the assumption that barriers in access to and navigating of healthcare for migrants can result in delays in cancer diagnosis. However, there may be alternative explanations for any observed difference in cancer stage distribution between migrants and non-migrants.

## Methods

### Search strategy

**Fig. 1:**
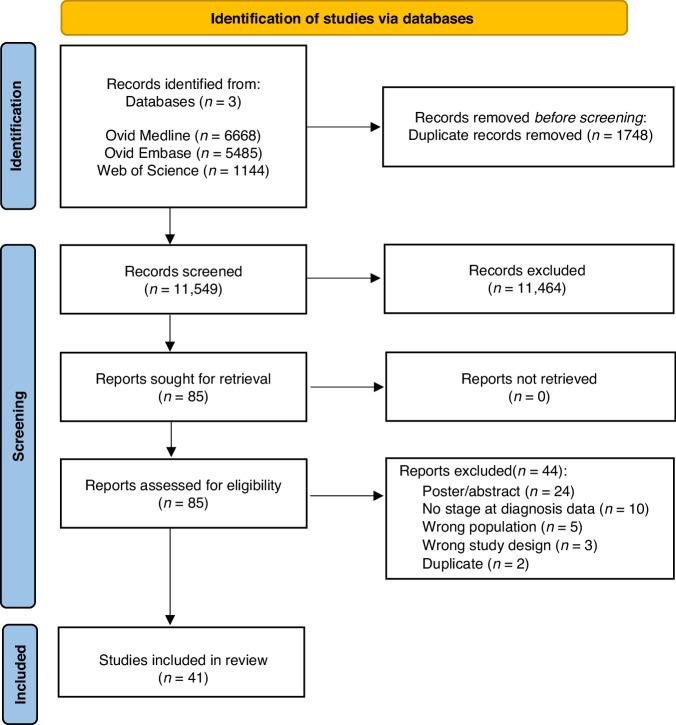
PRISMA Flow diagram [77]. The study selection processes are illustrated, covering the stages of identification, screening and inclusion of studies. Note that the use of *n* indicates the number of studies included at each stage.

Our systematic review and meta-analysis was conducted and reported in accordance with PRISMA guidelines (Fig. 1); a study protocol was registered with PROSPERO (CRD42023385332). Literature searches were performed in Ovid Embase, Ovid Medline, and Web of Science. A bibliographic search of the included studies was also conducted. Studies with terms related to ‘migrant’, ‘stage at diagnosis’ and ‘cancer’ were identified (see Supplementary Material for full search strategy). Studies were limited to those published from 1st January 2000 to 1st January 2023 and conducted in Organisation for Economic Co-operation and Development (OECD) countries, to improve their comparability. The search was restricted to studies indexed in English, but no restriction was applied with respect to the language of publication.

Eligible studies (i) included adult migrants, defined as ‘a person residing in a country different from one they were born in’, (ii) presented primary cancer stage at diagnosis data which was (iii) disaggregated by migration status (migrant or non-migrant). Editorials, posters, abstracts, and protocols were excluded. Studies were excluded if participants were temporary foreign visitors such as tourists; if the study reported on cancer without exploring diagnosis; if it discussed barriers and facilitators of diagnosis without comment on timeliness; or if diagnoses were for secondary cancers or solely identified via screening programmes. Screening only studies were excluded because screening represents a minority of cancer diagnosis (<7%), the barriers and facilitators are distinct, and reviews focussed on screening have already demonstrated inequality in cancer diagnosis [20, 25]. This study included studies where patients were symptomatic at presentation. After deduplicating citations, title/abstracts were screened by two of six independent reviewers (AHS, GF, HP, JH, PD, SA). All full texts that satisfied the eligibility criteria were retrieved and reviewed by two independent reviewers. Any disagreements between reviewers on study eligibility were discussed until consensus was reached. Rayyan, an online software platform (http://rayyan.qcri.org), was used to facilitate study screening and selection [26].

### Data extraction and analysis

Data extraction for each paper was performed by two independent reviewers from a pool of six reviewers (AHS, GF, HP, JH, PD, SA) using a standardised and piloted data extraction tool. To mitigate inter-rater variation, all six reviewers met to work through examples of screening, extraction and quality assessment. Disagreements were resolved by consensus. Once extracted, the stage at diagnosis data was dichotomised into “early” and “late”. Early stage at diagnosis was defined as either stage I and II or local, depending on the cancer stage classification system. Late stage at diagnosis was defined as either stage III and IV or regional/distant. Odds ratios (OR) were calculated for early stage at diagnosis comparing migrants to non-migrants.

For the meta-analysis, a random effects model was used to pool odds ratios (OR) using a restricted maximum-likelihood method (REML). Study heterogeneity was assessed using I^2^ statistics and Cochran’s Q test. A Leave-One-Out analysis was performed to assess what impact each study had on heterogeneity. Funnel plots were produced to assess publication bias. STATA^©^ 17 (StataCorp LLC, College Station, TX, USA) software was used to perform all pooled analyses and to produce forest and funnel plots.

Three or more study cohorts with data that could be dichotomised into early/late stage at diagnosis were required for pooled meta-analysis. For studies without suitable data, a narrative synthesis approach was used. This involved synthesising the data comparing cancer stage distribution between migrants and non-migrants and exploring relationships that might explain any observed difference [27]. Analyses were stratified by cancer type. We performed a priori defined subgroup analysis by migrant region of origin. Region of origin was classified based on the World Bank Classification system [28]. Similarly, subgroup analysis was conducted stratifying by study location whether inside or outside the USA, as a large proportion of the studies were USA based. Sensitivity analyses for missing data and using a local/non-local stage at diagnosis classification scheme were performed.

### Quality assessment

The Risk of Bias in Non-randomised Studies of Exposure (ROBINS-E) tool was applied independently to all studies by two from a pool of six reviewers (AHS, HP, JH, PD, SA) [29]. Study quality was assessed for the outcome of stage at diagnosis and rated from low to very high risk of bias across seven domains. Assessment ratings were compared, and disagreements were resolved by consensus. There was no formal assessment of inter-rater variation.

## Results

Our search strategy identified 11,549 articles. Of these, 85 articles were eligible for full-text screening. 41 papers met the inclusion criteria for this systematic review (Fig. 1). Of the 41 studies identified, 34 studies had suitable data for a meta-analysis of a two-group comparison on the dichotomised outcome of early vs late stage cancer at diagnosis.

**Fig. 2:**
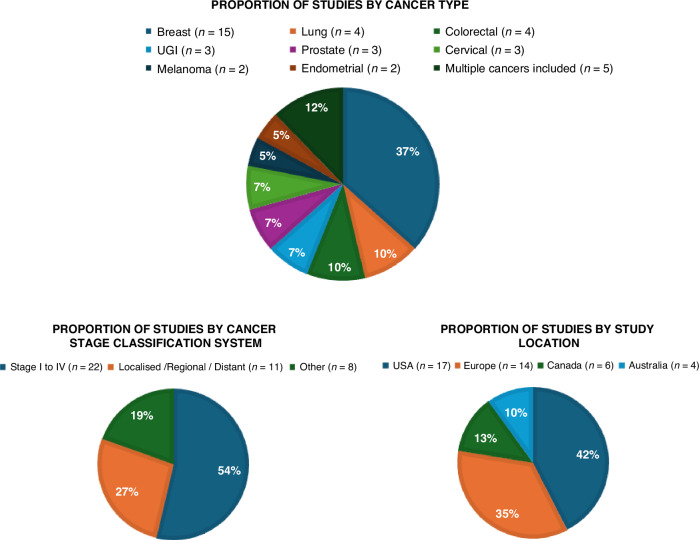
Study characteristics. Proportions of included studies according to cancer type, study location and cancer stage classification system.

Study characteristics are shown in Fig. 2, which highlights the variation by cancer type, study location, and cancer stage classification scheme. 26 of the studies used “foreign-born” as their definition of migrant, whilst the remaining 15 studies used other definitions that varied by specifying countries or regions of origin, or ethnicity.

### Cancer stage at diagnosis

#### All studies

**Fig. 3:**
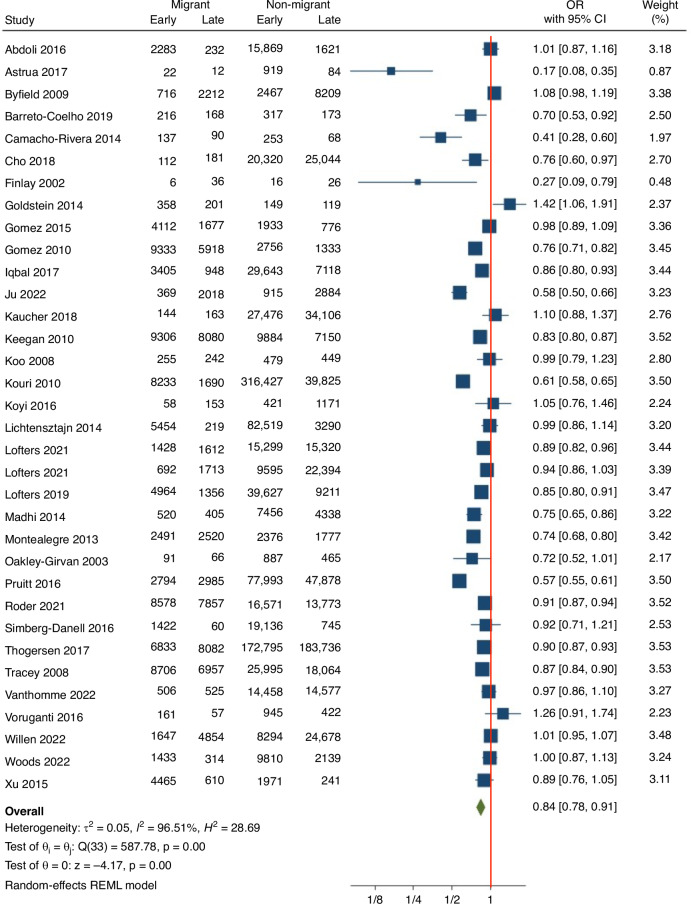
Forest plot of pooled Odds Ratio (OR) and 95% CIs for all studies, comparing early stage cancer at diagnosis, between migrants to non-migrants. Odds Ratio and 95% CI are presented by the central box and the horizontal line. The size of the box reflects the number of participants included in each study. The red line at OR = 1 indicates no effect.

The overall pooled result indicates that migrants were significantly less likely to be diagnosed with early stage cancer compared with native born participants (OR 0.84; 95% confidence interval (CI) 0.78–0.91) (Fig. 3). When stratified by study setting, in both the USA (*n* = 15) and non-USA (*n* = 19) based studies, migrants were significantly less likely to be diagnosed with early stage cancer compared with native born participants (Table 1). Of note, the heterogeneity was significantly reduced in non-USA based studies (I^2^ of 69% compared to 96% in those based in the USA). Further results were stratified by cancer site.

**Table 1:** Odds of early stage at diagnosis for migrants as compared to non-migrants, stratified by cancer type, migrant region of origin and study location. Results are presented as: OR (95% Confidence Interval), I^2^; number of studies.I^2^ is a measure of heterogeneity of the studies included in the meta-analysis.

Cancer type	Migrant region of origin						Study Location	
	East Asia & Pacific	Europe & Central Asia	Latin America & Caribbean	Middle East & North Africa	South Asia	Sub-Saharan Africa	USA	Outside USA
All	0.97 (0.87–1.09) 88%; 14	0.91 (0.81–1.02) 89%; 12	0.72 (0.63–0.82) 96%; 12	0.66 (0.25–1.80), 99%; 7	0.76 (0.65–0.89) 76%; 7	0.84 (0.73–0.96) 0%; 3	0.74 (0.65–0.84), 96%; 15	0.93 (0.89–0.96), 69%; 19
Breast	1.08 (0.90–1.30), 92%; 6	0.88 (0.78–0.99), 75%; 5	0.64 (0.56–0.75), 94%; 7	0.74 (0.58–0.94), 71%; 3	0.64 (0.57–0.72), 33%; 5	*	0.66 (0.55–0.78), 97%; 6	0.88 (0.86–0.91), 33%; 8
Lung	*	1.00 (0.88–1.14), 0%; 3	*	*	*	*	*	0.99 (0.94–1.04), 0%; 6
Colorectal	0.81 (0.64–1.02), 48%; 3	1.02 (0.86–1.20), 77%; 6	*	0.89 (0.55–1.43), 83%; 4	*	*	*	0.96 (0.89–1.05),45%; 6
Prostate	0.94 (0.84–1.04), 0%; 4	0.97 (0.70–1.33), 31%; 3	*	*	*	*	0.92 (0.81–1.04), 26%; 3	0.96 (0.75–1.23), 18%; 3

OR < 1.0 indicates that migrants are less likely to have early cancer stage at diagnosis compared to non-migrants.

North America not included due insufficient studies for analysis. * = insufficient studies for analysis.

#### Breast cancer

**Fig. 4:**
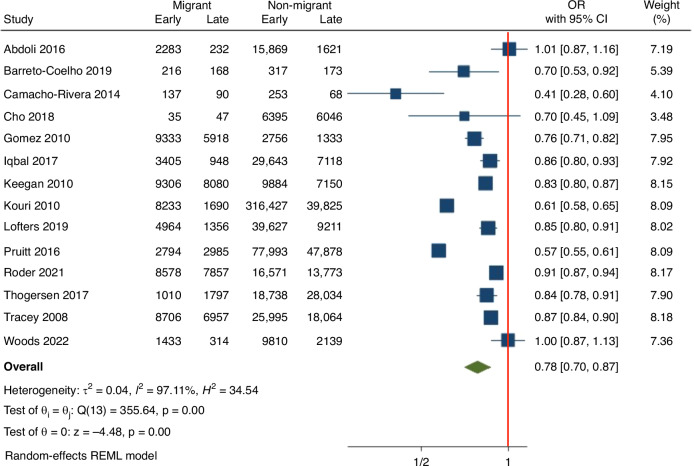
Forest plot of pooled Odds Ratio (OR) and 95% CIs for breast cancer studies comparing early stage cancer at diagnosis between migrants to non-migrants. Odds Ratio and 95% CI are presented by the central box and the horizontal line. The size of the box reflects the number of participants included in each study. The red line at OR = 1 indicates no effect.

In total, 18 studies included breast cancer diagnoses, of these, 14 studies were included in our meta-analysis [30–43]. The pooled result (Fig. 4; see Supplementary Material for remaining forest plots according to cancer type) indicates that migrants were significantly less likely to be diagnosed with early stage breast cancer (OR 0.78; 95% CI 0.70–0.87).

Subgroup analysis of breast cancer diagnoses by region of origin demonstrated that migrants from most regions were less likely to have early stage at diagnosis, although this association with not statistically significant for those from the East Asia and Pacific regions (Table 1). When stratified by study setting, in both the USA (*n* = 6) and non-USA (*n* = 8) based studies, migrants were significantly less likely to be diagnosed with early stage breast cancer (Table 1). Excluding studies from the USA significantly reduced heterogeneity (I^2^ reduced to 33% from 97%).

Narrative synthesis of studies not included in meta-analysis showed two studies also demonstrating the finding that migrants were less likely to be diagnosed with early cancer stage [44, 45]. Ziadeh et al. found this association remained after adjusting for age at diagnosis, year at diagnosis, marital status, SES and health insurance [44]. Norredam et al. also found that migrants were significantly more likely to have an unknown stage at diagnosis [45]. Unknown stage at diagnosis has been associated with worse outcomes previously [46]. Two studies, both based in Germany, found no difference in stage at diagnosis by migration status [47, 48]. Of note, Kaucher et al. focused on a unique migrant group from the former Soviet Union who are ethnic Germans, known as Spätaussiedler, who might be expected to have little observable difference from the non-migrant German population.

#### Lung cancer

All seven studies that included lung cancer diagnoses were eligible for meta-analysis. The pooled result indicates that there was no significant difference for early stage at diagnosis of lung cancer (OR 0.99; 0.94–1.03) with an I^2^ of 0%. Subgroup analysis by immigrant region of origin was only possible for migrants from Europe and Central Asia across three studies also demonstrated no significant difference (OR 1.00; 0.88–1.13) (Table 1). The same was found for the six studies conducted outside the USA (OR 0.99; 0.94–1.04) (Table 1).

#### Colorectal cancer

Six of the seven studies that included colorectal cancer diagnoses were suitable for meta-analysis, all were conducted outside the USA [33, 41, 47, 49–51]. The pooled result indicates that there was no significant difference for early stage at diagnosis of colorectal cancer (OR 0.97; 0.88–1.05) with an I^2^ of 45%. Dahlhaus et al. did not have suitable data for meta-analysis but reported that migrants consistently had later stage at diagnosis compared with native Germans [52]. Whilst the difference was not significant due to wide confidence intervals, this may be due to the small study size of 437 participants. Subgroup analysis of our meta-analysed studies showed no significant difference was found by region of origin (Table 1).

#### Upper gastrointestinal cancer

Five of the six studies that included Upper Gastrointestinal (UGI) cancer diagnoses, were suitable for meta-analysis [33, 41, 47, 53, 54]. The pooled result indicates that migrants were less likely to have an early stage of UGI cancer at diagnosis but a significant difference was not identified (OR 0.86; 0.64–1.16) with an I^2^ of 89%. Asokan et al. did not have data suitable for our meta-analysis but reported that there was no significant difference by migrant status for local vs non-local stage of oesophageal cancer at diagnosis [55].

#### Prostate cancer

All six studies on prostate cancer diagnoses were included in meta-analysis [33, 41, 47, 56–58]. The pooled result indicates that migrants were significantly less likely to be diagnosed with early stage prostate cancer (OR 0.92; 95% CI 0.85–0.99). When stratifying by migrant region of origin or by study setting the statistically significant difference did not remain (Table 1). Given the limited number of studies, – four or fewer for each analysis – this may suggest that this stratified meta-analysis was under-powered to detect a difference.

#### Cervical cancer

Four of the five studies on cervical cancer diagnoses were included in meta-analysis [41, 59–61]. The pooled result indicates that there was no statistically significant difference for early stage at diagnosis of cervical cancer (OR 0.97; 0.78–1.21) with high heterogeneity (I^2^ 90%). Norredam et al. pooled gynaecological cancers diagnosed in Denmark including cervical, endometrial, and ovarian cancers so did not have data suitable for our meta-analysis. They found that migrant women had decreased odds of being diagnosed with local stage but that it was non-significant, adjusting for match and age (OR 0.92 [95% CI 0.48–1.75]) [45].

### Melanoma

All three studies on melanoma diagnoses were suitable for meta-analysis and were conducted outside the USA [33, 62, 63]. The pooled result indicates migrants were less likely to have an early stage of melanoma at diagnosis but a significant difference was not identified due to notably wide confidence intervals (OR 0.50; 0.16–1.52) with an I^2^ of 87%. Whilst migrants were more likely to have late stage diagnosis across all three studies, it did not reach statistical significance with wide confidence intervals.

#### Endometrial cancer

Two studies looked at endometrial cancer diagnosis [64, 65]. Mahdi et al. found that immigrant Hispanic whites were less likely to be diagnosed with early stage endometrial cancer when compared to all USA-born individuals (unadjusted OR 0.75 (95% CI 0.65–0.86)) but there was no difference when compared specifically to USA-born Hispanic whites [65]. Svanvik et al. found immigrant women aged 50–74 years in Sweden did not have increased incidence rate of late-stage endometrial cancer relative to native counterparts [64].

#### Quality assessment

After quality assessment, two studies were assessed as having a very high risk of bias; 25 studies as having a high risk of bias and 14 studies had some concerns for bias (see Supplementary Material). The most common risks of bias were lack of adjustment for confounding, lack of a robust definition of the migrant population and missing data for either migrant status or stage at diagnosis. Studies commonly reported stage at diagnosis as a part of a descriptive analysis rather than as an adjusted outcome of a multivariable analysis.

**Fig. 5:**
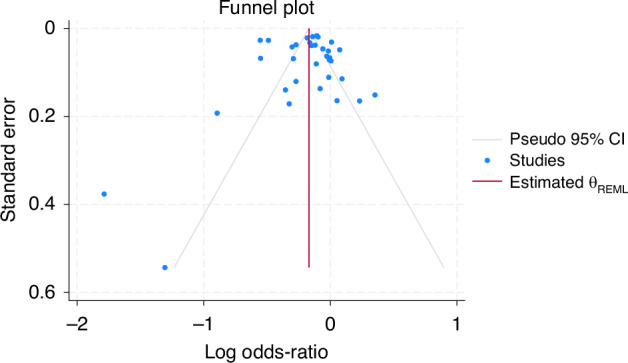
Funnel plot indicating an assessment of publication bias. This figure presents a funnel plot depicting the relationship between the log odds-ratio and its standard error across multiple studies to assess potential publication bias. This plot includes the studies, indicated by the blue dots, a pseudo 95% CI, indicated by the grey lines, and the estimated θ_REML_;indicating the overall effect size, represented by the central line.

The funnel plot shows a reasonably symmetrical distribution around the estimated effect size (Fig. 5), some slight asymmetry suggests potential publication bias in the selected studies, but this might be explained by the heterogeneity observed between the studies.

#### Sensitivity analyses

Overall, imputation analysis of missing data as either early or late stage resulted in no significant change in the interpretation of outcomes (see Supplementary Material). Two exceptions were: (a) in UGI cancer when imputing missing data as late stage changed the outcome from no significant difference to migrants being less likely to be diagnosed with early stage disease, which might indicate imputation added power to the analysis and (b) in prostate cancer when imputing missing data as late stage, when the interpretation change from migrants being less likely to have an early stage diagnosis to having no significant difference. A sensitivity analysis using a local vs non-local stage at diagnosis classification scheme did not change the interpretation of the outcome, nor did a sensitivity analysis that excluded the two studies rated at very high risk of bias.

## Discussion

### Summary of findings

To our knowledge, this is the first review to offer meta-analytical comparisons of stage at diagnosis between migrants and non-migrants across multiple cancers. Our results demonstrate that, overall, among cancer patients compared with non-migrants, migrants are less likely to be diagnosed with early stage disease. This was statistically significant for the overall results, as well as for breast and prostate cancer when stratified by cancer type. Across other cancer types, migrants were consistently less likely diagnosed with early stage cancer, although this difference was not significant. This may be in part due to the limited number of studies available for meta-analysis for certain cancer types, as demonstrated in UGI cancer when adding power in imputation resulted in the difference becoming statistically significant. Subgroup analysis found that although there was some variation, these findings were consistent across study locations and migrants’ region of origin.

### Strengths and Limitations

The strengths of this study include a prospectively published study protocol and a robust search strategy that searched across multiple cancer types allowing us to present a comprehensive overview of this topic. An expert panel of cancer and pathology experts informed the process of harmonising the stage at diagnosis classification across cancer types. Screening, data extraction and quality assessment were completed by two independent reviewers. Multiple sensitivity analyses were conducted to examine the impact of missing data and cancer stage classification scheme on the results.

This study has several limitations. Stage at diagnosis data was often not the primary outcome of the studies themselves and sometimes taken from unadjusted figures. Cancer stage classification is complex, varying across cancer types, classification systems and evolving over time. This changing case definition risks misclassification bias, which we mitigated by taking advice from our expert panel. Sensitivity analysis indicated that migrants remained less likely to have early stage at diagnosis across the different cancer classification systems.

There was significant heterogeneity across the studies. This was expected given the inclusion of heterogenous populations in different healthcare systems, presenting with a range of cancer types. Notably, studies conducted outside the USA consistently had lower heterogeneity than those conducted within the USA. This may be explained by the size of the USA is compared to the other included OECD countries with significant variation in the estimated 46 million migrants across the 50 states in terms of socio-economic demographics as well as disparities in how the fragmented healthcare systems interact with migrants. For example, the background and healthcare experience of the 10 million migrants living in California is likely to be quite distinct from the 20,000 migrants living in Wyoming. Greater heterogeneity in USA-based studies was also observed in Herbach’s study of disparities in breast cancer staging who attributed to marked differences in healthcare experience according to cultural background [66].

Across the studies there was significant amounts of missing data. However, most sensitivity analyses did not result in a change in direction of effect. Typically, migrants were more likely to have missing data than non-migrants, although this was not formally tested. To reduce publication bias, we searched all key databases as well as the grey literature and citation searching. The slight asymmetry observed in the funnel plot suggests a degree of publication bias might persist.

Whilst this study aimed to explore symptomatic diagnosis of cancer, some included studies did not disaggregate between symptomatic or screening-based diagnoses. Although screening accounts for <7% of all cancer diagnosis and <30% of breast cancer diagnoses specifically, inequalities in cancer screening uptake were likely echoed in our studies and it is possible that this accounts for some of the observed difference in stage at diagnosis for cancer types where screening programmes exist [25, 67].

### Comparison with existing literature

This study is consistent with the meta-analysis by Herbach et al. who found that migrants were significantly less liked to be diagnosed with localised stage breast cancer (OR 0.88, 95% CI 0.82–0.95) [66]. Similarly, our findings accord with the inequalities identified in the cancer screening literature, which has generally found that migrants are less likely to receive screening as compared to non-migrants [20, 68]. More broadly, across non-communicable diseases, migration is associated with interruption of healthcare and challenges to accessing healthcare during and following the migratory process with associated risk of delays in diagnosis and management [69]. Whilst studies of healthcare utilisation by migrants demonstrated increased rates of emergency department attendance and hospitalisation [70]. This could be explained by delays in diagnosis resulting in late stage presentation to healthcare services with advanced disease requiring more emergency care.

Ethnicity and migration status are highly interdependent variables as in particular settings certain ethnic groups are more likely to have a specific migration status. As such a degree of collinearity might be expected. However, a recent UK study showed limited evidence of ethnic differences in cancer diagnostic intervals [24]. This suggests potentially important differences on the influence of these two factors on cancer diagnosis.

### Implications of findings

Our findings highlight an inequality in cancer diagnosis between migrants and non-migrants across the OECD. Given the established literature on the patient-, provider-and system-related barriers in access to and navigation of healthcare for migrants, these are likely significant contributing factors. However, this was not explicitly tested in this study. There may also be other explanatory factors such as biological differences. For example, there is some evidence that migrants from “non-western” countries are more prone to infection-attributable cancers [71].

Differences in findings by cancer type highlight that there are different cancer-, patient-, clinician- and health system factors that influence the diagnostic pathway for each malignancy. Stage specific survival varies by cancer type. Breast cancer has a 100% age-standardised 1-year survival at stage one, compared to 66.6% at stage four; lung cancer has 91.9% survival at stage one but 23.9% at stage four; whilst prostate cancer has 100% survival at stage one compared to 89.6% at stage four [67]. Cancers that are less amenable to early diagnosis, such as lung cancer, are less likely to have a difference in stage at diagnosis between migrants and non-migrants. Conversely, cancers with a symptomatic early stage or those which can be identified by screening, including breast cancer, are more likely to have a difference. Furthermore, different presenting symptoms of cancer may have varying cultural sensitivities that impact presentation to healthcare services. For example, cultural taboos around breast examination, post-menopausal bleeding (indicative of endometrial cancer) or rectal bleeding (indicative of colorectal cancer) can provide a barrier to health-seeking [72]. These distinctions highlight the need for interventions tailored by cancer type and migrant group.

Differences between migrants also influences the diagnostic journey. Variations exists between migrant groups within regions, between regions and across the migration journey. Whilst evidence supporting the healthy migrant hypothesis suggests that in general, migrants to high-income countries have lower all-cause mortality than the non-migrant population, certain groups such as asylum seekers have increased mortality [73] and increased morbidity for certain conditions such as mental illness [74]. This review showed there was variation by region of origin of the migrant. This provides evidence for the idea that the cancer-risk profiles of migrants are determined by a complex mix of biological and socio-economic factors over their life course including their pre-migrant context [75].

### Recommendations for research, policy, and practice

Further research is required to understand potential mechanisms for differences in the cancer stage distribution between migrants and non-migrants and to investigate the factors that differentiate different cancer pathways and migrant typologies. Qualitative work is underway to explore the experiences of migrants in access to and navigating of healthcare on the cancer diagnosis pathway. Analysis of population-based electronic health records is needed to understand the potential impact of migrant-specific factors, such as age at migration, time since migration and requirement for translation services, on stage at diagnosis and cancer outcomes. This could also provide insight into routes to cancer diagnosis e.g., screening, emergency, or cancer referral, which is associated with cancer outcomes.

Tackling health inequalities is a major healthcare priority. Similarly, improving early cancer detection is a priority within cancer policy [76]. As policymakers develop strategies to improve cancer detection it is essential that they consider ways to mitigate inequalities in cancer diagnosis and look to explore evidence-based targeted interventions to reduce inequalities for migrant communities. Public oriented interventions can include health awareness campaigns and working with local communities. Healthcare professional oriented interventions can include tailored training programmes. These can inform improvements in clinical practice not only to reduce cancer inequities through culturally competent care but also improved data collection on migrant-specific determinants of health.

## Supplementary information

Supplementary material

## Data Availability

The data that support the findings of this study, not found in the article or supplementary material. are available from the corresponding author, AHS, upon reasonable request.
