# Supplementary material for: Comparing cancer stage at diagnosis between migrants and non-migrants: a meta-analysis
Source: Br J Cancer. 2024 Nov 12;132(2):158–67. doi: 10.1038/s41416-024-02896-0 (PMC11756392; doi:10.1038/s41416-024-02896-0)
Supplement: Supplementary file 1 — Supplementary material [file 41416_2024_2896_MOESM1_ESM.docx]

**Supplementary material**

**Search Strategy**

Ovid Embase and Medline

1. exp Neoplasms/

2. (cancer* or carcinoma* or neoplas* or malignan* or tumo?r*).ti,ab,kf.

3. 1 or 2

4. (diagnos$ or detect$ or consult$).ti,ab,kf.

5. ((prediagnos* or diagnos* or patient* or provider or system or doctor or physician or referral or primary care or investigation or clinic* or consult*) adj4 (delay* or interval* or timeliness or path? or pathway*)).ti,ab,kf.

6. exp "Early Detection of Cancer"/

7. (2WW or TWW or Two week wait).ti,ab,kf.

8. ((Urgent or rapid) adj5 (referral* or pathway*)).ti,ab,kf.

9. (time to diagnosis or time-to-diagnosis).ti,ab,kf.

10. (time to first presentation or time to first consultation).ti,ab,kf.

11. or/4-10

12. (migrat* or migrant* or refugee* or asylum seek* or ex-patriate* or (Foreign born or foreign-born) or immigra* or emigra*).ti,ab,kf.

13. exp Human Migration/

14. exp "Emigrants and Immigrants"/

15. exp "Transients and Migrants"/

16. exp Refugees/

17. or/12-16

18. (cell* adj5 migrat*).mp. or exp Cell Movement/

19. 17 not 18

20. 3 and 11 and 19

21. exp Animals/

22. exp Humans/

23. 21 not 22

24. 20 not 23

25. limit 24 to english language

26. limit 25 to yr="2000 -Current"

27. afghanistan/ or africa/ or africa, northern/ or africa, central/ or africa, eastern/ or "africa south of the sahara"/ or africa, southern/ or africa, western/ or albania/ or algeria/ or andorra/ or angola/ or "antigua and barbuda"/ or argentina/ or armenia/ or azerbaijan/ or bahamas/ or bahrain/ or bangladesh/ or barbados/ or belize/ or benin/ or bhutan/ or bolivia/ or borneo/ or "bosnia and herzegovina"/ or botswana/ or brazil/ or brunei/ or bulgaria/ or burkina faso/ or burundi/ or cabo verde/ or cambodia/ or cameroon/ or central african republic/ or chad/ or exp china/ or comoros/ or congo/ or cote d'ivoire/ or croatia/ or cuba/ or "democratic republic of the congo"/ or cyprus/ or djibouti/ or dominica/ or dominican republic/ or ecuador/ or egypt/ or el salvador/ or equatorial guinea/ or eritrea/ or eswatini/ or ethiopia/ or fiji/ or gabon/ or gambia/ or "georgia (republic)"/ or ghana/ or grenada/ or guatemala/ or guinea/ or guinea-bissau/ or guyana/ or haiti/ or honduras/ or independent state of samoa/ or exp india/ or indian ocean islands/ or indochina/ or indonesia/ or iran/ or iraq/ or jamaica/ or jordan/ or kazakhstan/ or kenya/ or kosovo/ or kuwait/ or kyrgyzstan/ or laos/ or lebanon/ or liechtenstein/ or lesotho/ or liberia/ or libya/ or madagascar/ or malaysia/ or malawi/ or mali/ or malta/ or mauritania/ or mauritius/ or mekong valley/ or melanesia/ or micronesia/ or monaco/ or mongolia/ or montenegro/ or morocco/ or mozambique/ or myanmar/ or namibia/ or nepal/ or nicaragua/ or niger/ or nigeria/ or oman/ or pakistan/ or palau/ or exp panama/ or papua new guinea/ or paraguay/ or peru/ or philippines/ or qatar/ or "republic of belarus"/ or "republic of north macedonia"/ or romania/ or exp russia/ or rwanda/ or "saint kitts and nevis"/ or saint lucia/ or "saint vincent and the grenadines"/ or "sao tome and principe"/ or saudi arabia/ or serbia/ or sierra leone/ or senegal/ or seychelles/ or singapore/ or somalia/ or south africa/ or south sudan/ or sri lanka/ or sudan/ or suriname/ or syria/ or taiwan/ or tajikistan/ or tanzania/ or thailand/ or timor-leste/ or togo/ or tonga/ or "trinidad and tobago"/ or tunisia/ or turkmenistan/ or uganda/ or ukraine/ or united arab emirates/ or uruguay/ or uzbekistan/ or vanuatu/ or venezuela/ or vietnam/ or west indies/ or yemen/ or zambia/ or zimbabwe/

28. "Organisation for Economic Co-Operation and Development"/

29. australasia/ or exp australia/ or austria/ or baltic states/ or belgium/ or exp canada/ or chile/ or colombia/ or costa rica/ or czech republic/ or exp denmark/ or estonia/ or europe/ or finland/ or exp france/ or exp germany/ or greece/ or hungary/ or iceland/ or ireland/ or israel/ or exp italy/ or exp japan/ or korea/ or latvia/ or lithuania/ or luxembourg/ or mexico/ or netherlands/ or new zealand/ or north america/ or exp norway/ or poland/ or portugal/ or exp "republic of korea"/ or "scandinavian and nordic countries"/ or slovakia/ or slovenia/ or spain/ or sweden/ or switzerland/ or turkey/ or exp united kingdom/ or exp united states/

30. European Union/

31. Developed Countries/

32. or/28-31

33. 27 not 32

34. 26 not 33

**Summary of study characteristics:**

| **First author, Year Published** | **Study design** | **Study period** | **Study setting** | **Cancer type** | **Migrant definition** | **Non-migrant definition** | **Stage at diagnosis classification system** | **Study sample size** |
| --- | --- | --- | --- | --- | --- | --- | --- | --- |
| **Abdoli 2016** | Retrospective Cohort study | 2004-2009 | Sweden | Breast | Foreign-born. Excluded foreign-born breast cancer patients that had resided in Sweden for less than 5 years (274 cases) before diagnosis. | Sweden-born | TNM classification | 35,542 |
| **Asokan 2020** | Retrospective cohort study | January 2003 - August 2018 | Boston, USA | Oesophageal | Country of origin | Born in USA | Clinical staging I-IV; divided into operable (I-III) and non-operable (IV) | 266 |
| **Astrua 2017** | A single-centre database retrospective case-control study | January 2002 - December 2015 | northern Italy | Melanoma | Patients whose country of origin is in Eastern Europe | A cohort of 1,003 melanoma patients selected from a total of 372,190 Italian patients who underwent a dermatological evaluation during the same time period. | Stage I-IV, also divided into early (I+II) and late (III+IV) | 1052 |
| **Byfield 2009** | Retrospective cohort study | 1992-2005 | USA | Gastric | Foreign-born Asian and Pacific Islanders | USA-born. They did not examine birthplace among non-Hispanic white (NHW) patients, because it is not well documented in medical records for white patients, and US Census data indicate that only 3.9% of NHW individuals in the United States are foreign-born | SEER staging: local, regional, and distant | 16553 |
| **Barreto-Coelho 2019** | Retrospective cohort study | 2006-2017 | Miami, USA | Breast | Caribbean-born, of black ethnicity | USA-born, of black ethnicity | Stage I-IV, also divided into early (I+II) and late (III+IV) | 1131 |
| **Camacho-Rivera 2014** | Retrospective cohort study | 2000-2010 | New York, USA | Breast | Foreign born (born outside the USA) | Born in the USA | AJCC Seventh Edition TNM criteria | 1,097 |
| **Cho 2018** | Retrospective, population-based cohort study | 1990-2009 | Saarland, Germany | lung, colorectal, breast, prostate, malignant melanoma, stomach | re-settlers from the former Soviet Union who migrated to the federal state of the Saarland during the period 1990 through 2005 | general population or autochthonous population of the Saarland describe all inhabitants of the federal state excluding the population of the cohort | TNM classification: Stage I-IV. Condensed into Local / Advanced / Missing |  |
| **Dahlhaus 2018** | A cross-sectional analysis | - | Frankfurt am Main, Germany | Colorectal | If the patient and/or at least 1 of his/her parents was born outside Germany, he/she was considered to have a migration background | Patients that were born in Germany and whose parents were also born in Germany were classified as non- migrants. | Union for International Cancer Control (UICC) stage. stage was dichotomized into I versus II–IV. Patients with UICC stage 0 were excluded from analysis. | 444 |
| **Finlay 2002** | Retrospective case-control study | January 1, 1992, to December 31, 1996 | A tertiary level care hospital providing all levels of medical care to the local Asian population in the Boston area | Lung | An Asian patient was defined as one who was born in the continent of Asia and had immigrated to the United States | Each Asian patient with lung cancer (case) was matched for age and sex with a non-Asian patient with lung cancer who also received a diagnosis of lung cancer in the same 5-year period (control). To reduce selection bias, each case was randomly matched using a computerized randomization program with one of five potential controls identified by the matching process. | TNM classification: Stage I-IV | 84 |
| **Gomez 2015** | A population-based database linkage study | 1994-2009 | California, USA | Cervical | Birthplace. They used birthplace information from the registry (available for 77% of cases), or if missing, by a validated method using the first 5 digits of the patient’s social security number (SSN) to determine the year the SSN was issued. For 5.4% of cases (n = 497), SSNs were unavailable or invalid, and nativity was randomly assigned based on the distribution of Hispanic cases by nativity. | Hispanic women, born in the USA | American Joint Committee on Cancer stage I vs stages II-IV | 9219 |
| **Gomez 2010** | A population-based database linkage study | January 1988 - December 2005 | California, USA | Breast | Migrant status determined by birthplace information from registry data, which was available for 73% of patients (70% from hospital medical records and 3% from death certificates). For the 26% of patients with unknown birthplace, immigration status was imputed from year of receiving a social security number (SSN). For the 1.7% of records with missing or invalid SSNs, they assigned immigrant status according to the ethnicity– gender–age nativity distribution of the overall sample. | Patients with Asian ethnicity, born in the USA | Localised/Regional/Remote/Unknown | 20,747 |
| **Iqbal 2017** | Population based, cross sectional | 2007-2012 | Canada | Breast | Listed on IRCC-PR database = Born outside of Canada and become resident of Ontario since 1985 | Not listed on IRCC-PR database | Stage I, II, III, IV (I vs II-IV in analysis) | 41,213 |
| **Ju 2022** | Retrospective cohort study | 2004-2017 | Texas, US | Stomach | Foreign born. | US born Hispanic | Localised, Regional, Metastatic (SEER) | 6,186 |
| **Kaucher 2018** | Retrospective cohort study | 1990-2001 - AMIN, 1994-2013 FCR, AMOR 1990-2005 | Münster, Germany | histologically confirmed, primary malignant tumours excluding melanoma – sub-analysis: colorectal, lung, prostate, breast, stomach | ethnic Germans who mainly immigrated from the Russian federation and other countries of the former Soviet Union after its collapse in 1989 | Munster general population with re-settler cohort removed | Local vs advanced based on T classification (size) and separately NM classification | AMIN - 32972, AMOR - 59390 |
| **Keegan 2010** | Retrospective cohort study | 1988-2005 | USA - California | Breast | Foreign born - Nativity was based on registry data or, if missing - (25.6%) imputed from case Social Security number, or randomly assigned if no SSN (2.2%). Sensitivity analysis done to compare if excluded and bias was introduced. | US born | local, regional, distant, missing | 37,695 |
| **Koo 2008** | Prospective cohort study | January 1, 1997, to December 31, 2004 | Sydney, Australia | Colorectal | Born outside of Australia | Australian born | Dukes staging | 1,496 |
| **Kouri 2010** | population-based, retrospective cohort study | 1988-2005 | USA | Breast | Hispanic - US born or foreign born | US born Hispanic or US born non-Hispanic white | Stage I-IV or unstaged | 403,325 |
| **Koyi 2016** | Retrospective cohort study | 1  January 2003 to 31 December 2008 | Sweden, Stockholm | Lung (non-small cell) | Foreign born. | Native born | Stage I-IV | 1,803 |
| **Kuehnle 2021** | Prospective, cross sectional study | 2012-2016 | Germany, Lower Saxony | Breast | First-generation immigrants are immigrants that immigrated to Germany themselves. Second-generation immigrants are children of at least one parent with a history of immigration | Natives are people that are born in Germany, have the German citizenship from date of birth, and whose parents are both born in Germany and have the German citizenship from date of birth. | UICC | 2,145 |
| **Latif 2015** |  | 2002-2009 | Norway | Breast | Born in Pakistan, Somalia, or Sri Lanka | Ethnic Norwegian women' were matched to migrant women who had surgery at same hospital on same day by same surgeon | Stage 2&3 and locally advanced - seems to use these interchangeably in the results section. I am very confused | 161 |
| **Lichtensztajn 2014** | Retrospective cohort study | 2004-2010 | California USA | Prostate | Foreign born Asian men | US born | Advanced stage (T3+) vs T2b,c. and Early Stage (T1–T2a) vs T2b,c,NOS | 90,845 |
| **Lofters 2021 (Colorectal)** | Population based retrospective cohort study | 2012-2017 | Canada | Colorectal | Immigration, Refugees and Citizenship Canada - Permanent Resident (IRCC-PR) dataset, which identifies Ontario immigrants who arrived in Canada from 1985 onward | Long-term residents - people not in IRCC-PR database i.e. arrived prior to 1985. There have lived in Ontario for at least 32 years (may be immigrants or non-immigrants) | Stage I-IV, divided into early (I+II) and late (III+IV) | 37,717 |
| **Lofters 2021 (Lung)** | Population based retrospective cohort study | 1 April 2012 and 31 March  2017 | Ontario province, Canada | Lung | Immigration, Refugees and Citizenship Canada - Permanent Resident (IRCC-PR) dataset, which identifies Ontario immigrants who arrived in Canada from 1985 onward | Long-term residents - people not in IRCC-PR database i.e. arrived prior to 1985. There have lived in Ontario for at least 32 years (may be immigrants or non-immigrants) | Stage I-IV, divided into early (I+II) and late (III+IV) | 38,788 |
| **Lofters 2019** | Retrospective cohort study | January 1, 2007 and December 31, 2011 | Canada | Breast | Immigration, Refugees and Citizenship Canada - Permanent Resident (IRCC-PR) dataset, which identifies Ontario immigrants who arrived in Canada from 1985 onward | Long-term residents - people not in IRCC-PR database ie. arrived prior to 1985. There have lived in Ontario for at least 32 years (may be immigrants or non-immigrants) | Stage I-IV, divided into early (I+II) and late (III+IV) | 61149 |
| **Mahdi 2014** | Retrospective cohort study | 1988-2009 | USA | Endometrial | Hispanic White born outside the USA | Hispanic white born in the USA | Stage I-IV, divided into early (I+II) and late (III+IV) | 1422 |
| **Montealegre 2013** | Retrospective population based cohort study | January 1,  1988 and December 31, 2008 | USA | Cervical | Foreign-born cases included those born in Latin America and Caribbean. To impute the missing values for nativity, a new regression model was simulated using the posterior predictive distribution of parameters based on the fitted regression coefficients. The process was repeated in 20 imputation datasets. The imputation strategy was validated in a random sample of 20 % of the observed data with known nativity status. | Born in USA | SEER: Early (local), late (distant and regional) | 9164 |
| **Norredam 2008** | Register-based cohort study | 1 January 1993 to 31 December 2002 | Honolulu (HI), Los Angeles (LA), San Francisco (SF), and Vancouver (BC). | All sites, sub-analysis of breast and gynaecological cancers | Women from Eastern Europe, Middle East and North Africa who obtained a residence permit through the Danish Immigration Service | Women born in Denmark | Local/Non-Local/Unknown | 1,877 |
| **Oakley-Girvan 2003** | Multicentre population-based case-control study | January 1, 1987, and December  31, 1991 |  | Prostate | Foreign-born - limited to foreign-born Asian Americans | Not foreign born. They did not classify African Americans and Whites according to place of birth, because virtually all were born in North America | Localised/Regional/Distant/Unknown | 1509 |
| **Pruitt 2016** | Retrospective cohort study | 1995-2009 | Texas, USA | Breast | Foreign born (born outside the USA). Only the Hispanic group disaggregated by birthplace. Non-Hispanic Whites presumed 'native-born' | Non-Hispanic White or Hispanic US-born | (SEER) summary stage (in situ, local, regional, distant, unstaged) | 166254 |
| **Roder 2021** | Retrospective cohort study | 2003-2016 | New South Wales, Australia | Breast | Born outside of Australia | Born in Australia | Localised/Regional/Distant/Unknown | 46,779 |
| **Simberg-Danell 2016** | Nationwide, population-based cohort study | January 1st, 1990- December 31st, 2007 | Sweden | Cutaneous malignant melanoma | Born outside of Sweden - first or second generation | country of birth - Sweden | I, II, III, IV, clinical stage unknown | 27235 |
| **Svanvik 2019** | Registry-based cross-sectional analysis | 1 January 1995 - 31 December 2016 | Western Sweden Healthcare Region | Endometrial (Endometrioid and Non-endometrioid) | Foreign-born | / | FIGO 2009 guidelines | 5,833 |
| **Thøgersen 2017** | nationwide, population-based cohort study | 1 January 1990 -31 December 2014 | Norway | Seven cancer sites were analysed (breast, cervix, colorectal, liver, lung and trachea, prostate, and stomach). | individuals born abroad whose parents were also born abroad | persons born in Norway, with at least one Norwegian-born parent. Persons born in Norway with two immigrant parents were defined as second-generation immigrants and were excluded from the analyses (n 5 808). Undocumented immigrants are not identifiable in the registries. | Localised/Regional/Distant/Unknown | 449,645 |
| **Tracey 2008** | Retrospective cohort study | 1980-2003 | Australia | breast | country of birth | Australian born / unknown | local, regional, distant | 59,731 |
| **Vanthomme 2022** | Retrospective cohort study | 2004-2013 | Belgium | Colorectal | only Italian, Turkish and Moroccan immigrants who migrated themselves to Belgium (i.e. first generation immigrants) and excluded those who immigrated only recently to Belgium (in 1991 or later). | "native Belgians" | Stage I-IV, divided into early (I+II) and late (III+IV) | 2,444,082 |
| **Voruganti 2016** | Retrospective matched cohort study | 2010-2014 | Ontario, Canada | Cervical | people who had a landed immigrant or permanent resident status at any time from 1985 to 2014 | those not identified in the Permanent Resident Database | late (II–IV) and early (I) | 2508 |
| **Willen 2022** | Population based, retrospective cohort study | 2002-2016 | Sweden | Lung  (non-small cell) | country/region of birth (we used Nordic and non-Nordic as immigrants in our analysis) | patients born in Sweden | IA-IIB, IIIA, IIIB-IV, missing (we have grouped 1a-2b as early and 3a/3b-4 as late) | 40,075 |
| **Woods 2022** | Population based, retrospective cohort study | 1 January 2010 - 31 Dec 2014 | British Columbia, Canada | Breast | Not defined - appears to be based on country of birth | non-immigrant - not defined | Data collected on Stage I, II, III, IV but analyses carried out on early stage (stage 1) and late stage (II to IV) | 1,342,317 |
| **Xu 2014** | Population based, retrospective cohort study | 2004-2009 | USA | Prostate | birthplace | US-born Asians | Gleason score / T classification - T0 - T2C; T3; unknown | 7824 |
| **Ziadeh 2018** | Population based, retrospective cohort study | January 1, 1988 - December 31, 2013 | California, USA | Breast | If the patient had a Middle Eastern [ME] last name , did not have a Hispanic or Asian last name, and was born in one of the Middle Eastern countries, she was considered a first-generation ME immigrant. | Non-Hispanic white [NHW]: did not have an ME or Hispanic or Asian last name and was identified as white in the CCR data set, she was considered NHW in our analysis. For our analysis we have combined NHW and second or subsequent generation immigrants as the control group. | SEER: In situ, localised, non-localised. non-localised tumours - regional and distant . | 352,013 |

**Risk of Bias in Non-randomised Studies of Exposure (ROBINS-E) assessment**

| **Table A: Risk of Bias in Non-randomised Studies of Exposure (ROBINS-E) assessment** | | | | | | | | | | |
| --- | --- | --- | --- | --- | --- | --- | --- | --- | --- | --- |
| **First author, year** | **Overall Risk of Bias** | **Confounding** | **Measurement of the exposure** | **Selection Bias** | **Post-exposure interventions** | **Missing data** | **Measurement of the outcome** | **Reporting bias** | **Comment** |  |
| **Abdoli 2016** | High | Low | Low | Some concerns | N/A | High | Low | Low | Significant missing data with 43% missing stage at diagnosis. |  |
| **Asokan 2020** | High | High | Some concerns | Some concerns | Low | Some concerns | Low | Low | Small sample size with missing data and no adjusting for confounders. |  |
| **Astrua 2017** | High | High | High | Some concerns | Low | Low | Low | Low | Small sample size with no adjusting for confounders. |  |
| **Byfield 2009** | High | Low | Low | Low | Low | High | Low | Low | Missing migrant status data, although no impact on sensitivity analysis. Significant missing stage data with no sensitivity analysis. |  |
| **Barreto-Coelho 2019** | High | High | Some concerns | Some concerns | N/A | Very High | Low | Low | 25% missing stage data and missing migrant status data. |  |
| **Camacho-Rivera 2014** | High | High | Low | Some concerns | N/A | High | Low | Low | ~38% missing migrant status data and no adjusting for confounders. |  |
| **Cho 2018** | High | Low | High | Low | Low | Low | Low | Low | Risk of non-differential misclassification between migrants and non-migrants. |  |
| **Dahlhaus 2018** | Some concerns | Some concerns | Some concerns | Some concerns | N/A | Low | Some concerns | Low | Some concerns that migrant definition might include a small number of second generation-migrants. |  |
| **Finlay 2002** | High | Some concerns | High | Some concerns | Low | Low | Low | Low | Small sample size with some concerns of referral bias and limited adjustment for confounders. |  |
| **Gomez 2015** | Some concerns | Some concerns | Low | Some concerns | Low | Some concerns | Low | Low |  |  |
| **Gomez 2010** | High | High | Low | Some concerns | Low | High | Low | Low | Missing migrant status and stage data with no adjustment for confounding. |  |
| **Iqbal 2017** | Some concerns | Low | Some concerns | Low | Low | Low | Low | Low | Excluded migrants arriving prior to 1985 |  |
| **Ju 2022** | High | High | Low | Some concerns | Low | Some concerns | Low | Low | 28% missing migrant status with no adjusting for confounders |  |
| **Kaucher 2018** | High | High | Some concerns | Some concerns | Low | High | Some concerns | Low | Up to 40% missing stage data, poorly defined exposure, and partial adjustment for confounding. |  |
| **Keegan 2010** | Some concerns | Some concerns | Low | Low | Low | Some concerns | Low | Low | Some missing migrant status data. |  |
| **Koo 2008** | High | High | Low | Some concerns | Low | Low | Low | Some concerns | No adjustment for confounding. |  |
| **Kouri 2010** | High | High | Very high | Low | Low | High | Low | Low | 38% missing migrant status data with partial adjustment for confounding. |  |
| **Koyi 2016** | High | High | Some concerns | Low | Low | Low | Low | Some concerns | No adjustment for confounding. |  |
| **Kuehnle 2021** | Very high | Very high | Low | Some concerns | N/A | Low | Low | High | Stage at diagnosis results not shown, only summarised. Unclear what statistical testing was done or if adjusted for confounding. |  |
| **Latif 2015** | Very high | Very high | High | Some concerns | N/A | Low | High | High | No statistical analysis presented and no adjustment for confounding. |  |
| **Lichtensztajn 2014** | High | Low | Some concerns | Some concerns | Low | High | Low | Low | 38% missing migrant status. Missing stage data. |  |
| **Lofters 2021 (Colorectal)** | Some concerns | Some concerns | Some concerns | Low | N/A | Some concerns | Low | Some concerns | Migrant definition not robust and did not adjust for ethnicity. |  |
| **Lofters 2021 (Lung)** | Some concerns | Some concerns | Some concerns | Some concerns | N/A | Some concerns | Low | Some concerns | Migrant definition not robust and did not adjust for ethnicity or smoking status. |  |
| **Lofters 2019** | Some concerns | Some concerns | Some concerns | Low | N/A | Some concerns | Low | Low | Migrant definition not robust and did not adjust for ethnicity. |  |
| **Mahdi 2014** | High | Very high | Low | Some concerns | N/A | Some concerns | Low | Low | No adjustment for confounding. |  |
| **Montealegre 2013** | High | High | Some concerns | Some concerns | N/A | Some concerns | Some concerns | Low | Migrant status imputed for 31% and partial adjustment for confounding. |  |
| **Norredam 2008** | High | Some concerns | Low | Some concerns | Low | Very high | Low | Low | Missing stage data with partial adjustment for confounding. |  |
| **Oakley-Girvan 2003** | High | Low | High | High | Low | Some concerns | Low | Low | Risk of selection bias as migrant participants limited to Asian-American ethnicity. |  |
| **Pruitt 2016** | High | High | High | Some concerns | Low | High | Low | Low | Risk of misclassification, significant missing data despite imputation analyses and no adjustment for confounding. |  |
| **Roder 2021** | Some concerns | Some concerns | Low | Some concerns | Low | Some concerns | Low | Low | Some missing stage data. |  |
| **Simberg-Danell 2016** | Some concerns | Low | Low | Some concerns | Low | Some concerns | Low | Low | No time since immigration date data. |  |
| **Svanvik 2019** | Some concerns | Low | Low | Low | Low | Some concerns | Low | Low | Some missing stage data. |  |
| **Thøgersen 2017** | Some concerns | Low | Low | Low | Low | Some concerns | Low | Some concerns | Some missing stage data. |  |
| **Tracey 2008** | Some concerns | Low | Some concerns | Low | Low | Low | Low | Low | If unknown country of birth assumed to be native Australian. |  |
| **Vanthomme 2022** | High | High | Some concerns | Some concerns | Low | Some concerns | Low | Low | ~10% missing stage data and no adjustment for confounding. |  |
| **Voruganti 2016** | High | Some concerns | Some concerns | Some concerns | Low | High | Low | Low | 36% missing stage data and partial adjustment for confounding. |  |
| **Willen 2022** | High | High | Low | Some concerns | Low | Low | Low | Low | No adjustment for confounding. |  |
| **Woods 2022** | Some concerns | Some concerns | Some concerns | Some concerns | Low | Low | Low | Low | unable to track migrants who came to Canada via different province. |  |
| **Xu 2014** | High | High | Low | Some concerns | Low | High | Low | Some concerns | No adjustment for confounding. |  |
| **Ziadeh 2018** | Some concerns | Some concerns | Some concerns | Some concerns | Low | Low | Low | Low | Migrant definition not robust and partial adjustment for confounding. |  |

**Forest plots**

**
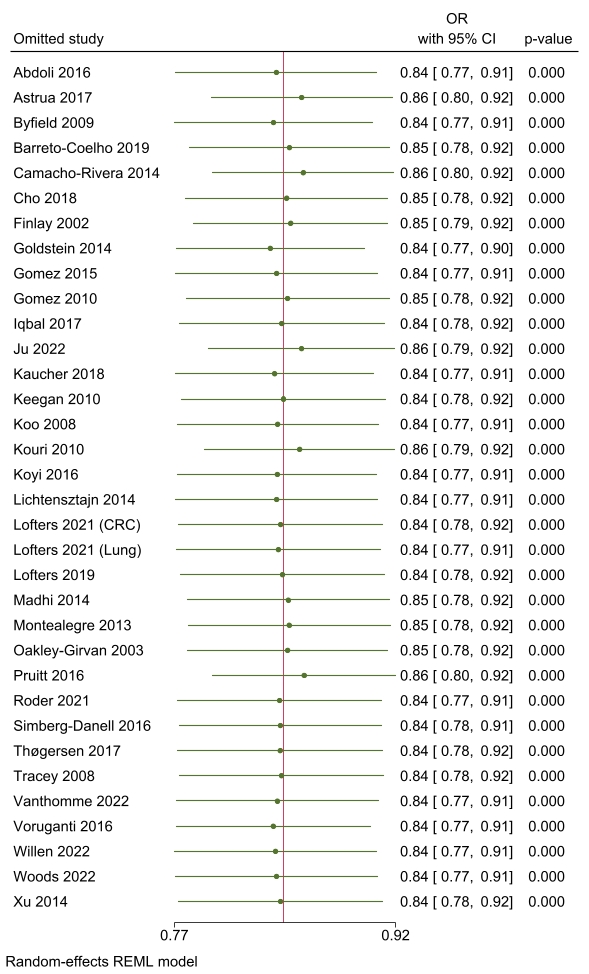
**

**Figure 1:** Leave-one-out forest plot of all studies comparing early stage cancer at diagnosis between migrants to non-migrants.

**
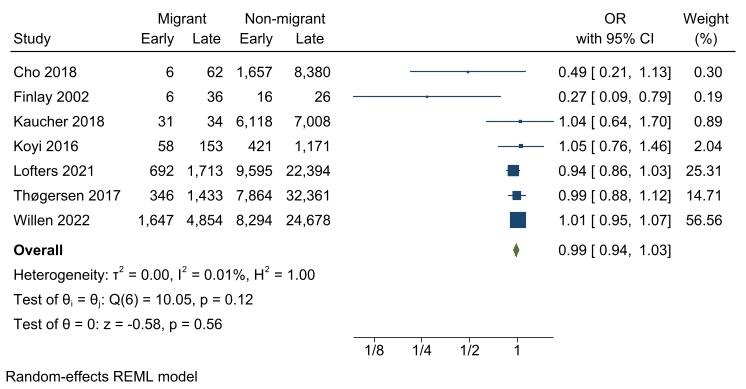
**

**Figure 2:** Forest plot of pooled Odds Ratio (OR) and 95% CIs for lung cancer studies, comparing early stage cancer at diagnosis between migrants to non-migrants.

**
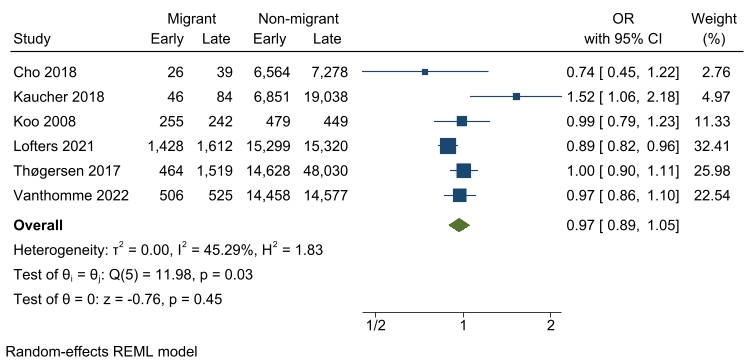
**

**Figure 3:** Forest plot of colorectal cancer studies pooled, comparing early stage cancer at diagnosis between migrants to non-migrants.

**
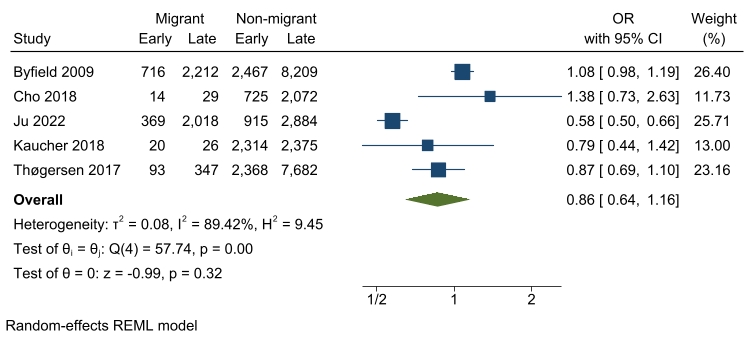
**

**Figure 4**: Forest plot of UGI cancer studies pooled, comparing early stage cancer at diagnosis between migrants to non-migrants.

**
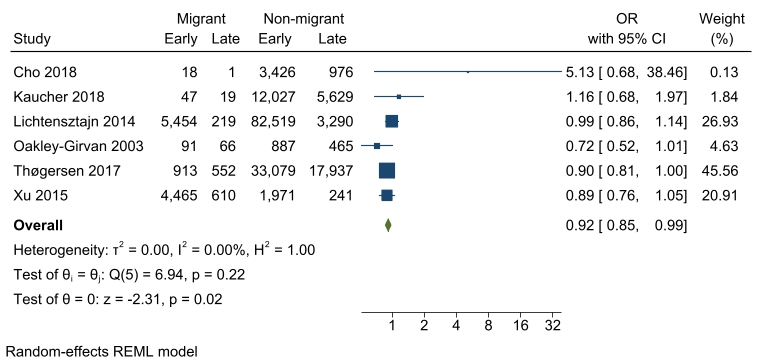
**

**Figure 5:** Forest plot of prostate cancer studies pooled, comparing early stage cancer at diagnosis between migrants to non-migrants.


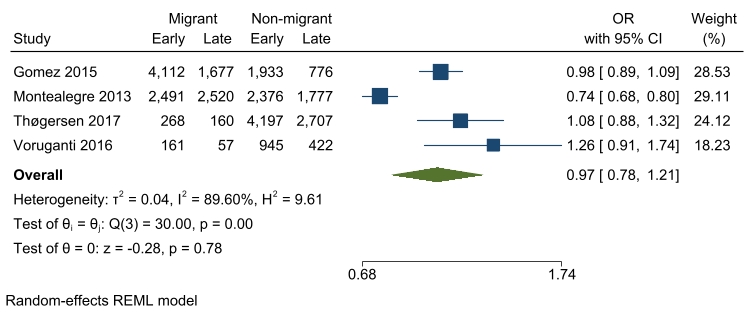


**Figure 6:** Forest plot of cervical cancer studies pooled, comparing early stage cancer at diagnosis between migrants to non-migrants.


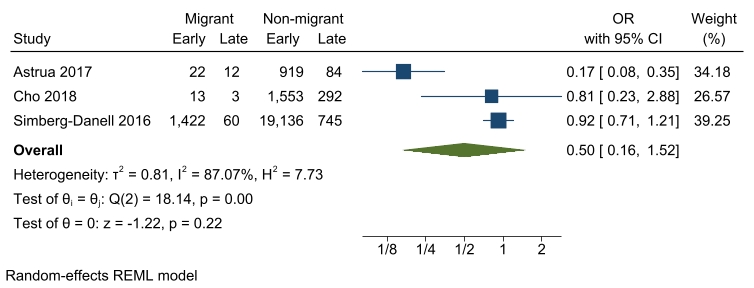


**Figure 7:** Forest plot of melanoma studies pooled, comparing early stage cancer at diagnosis between migrants to non-migrants.


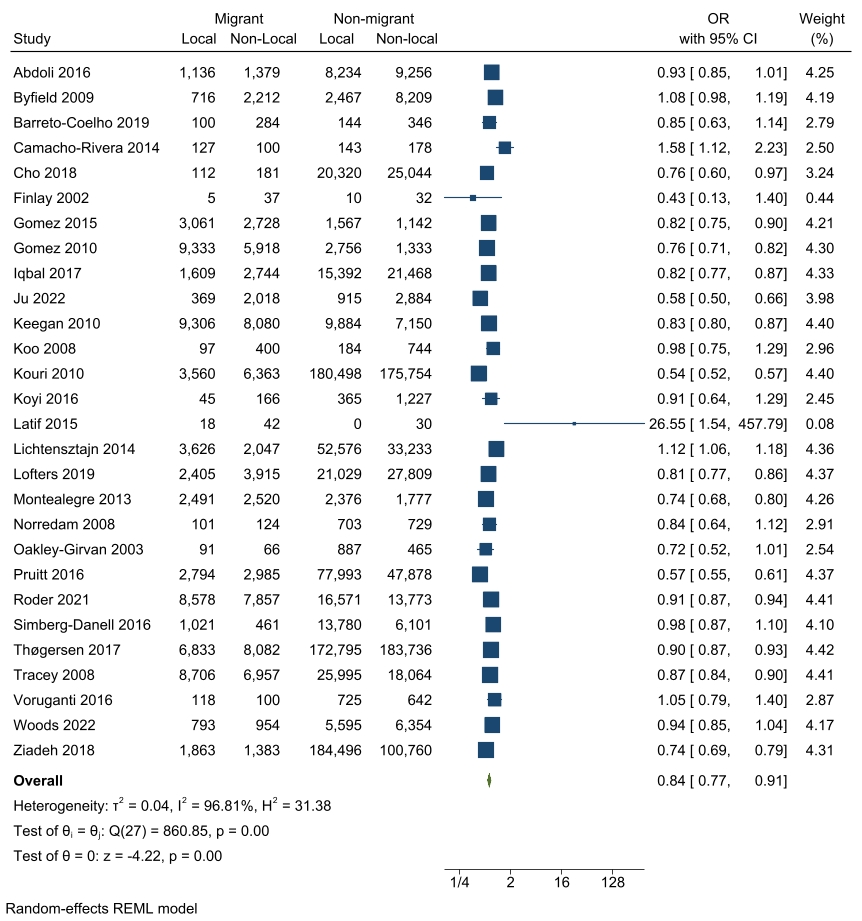


**Figure 8:** Forest plot of all studies pooled using local vs non-local classification, comparing local stage cancer at diagnosis between migrants to non-migrants.


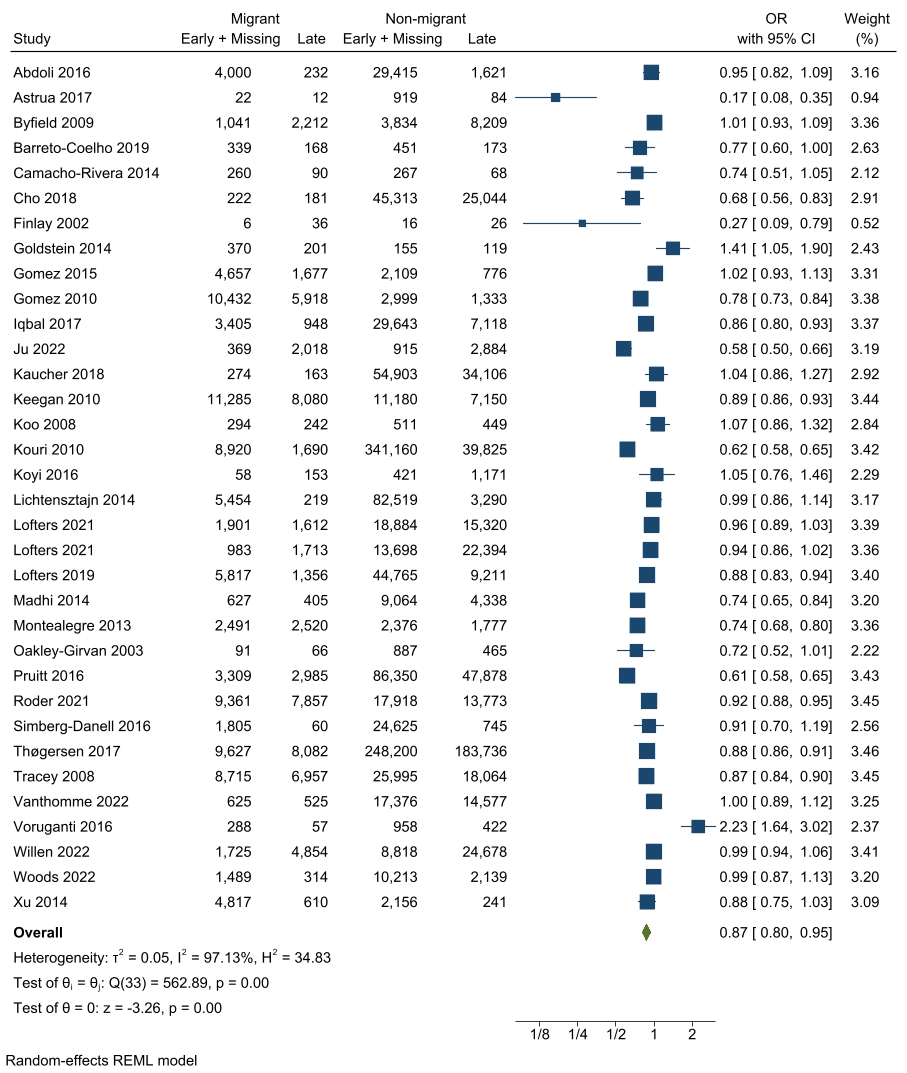


**Figure 9:** Forest plot of all studies pooled with missing data included as early stage, comparing early stage cancer at diagnosis between migrants to non-migrants.


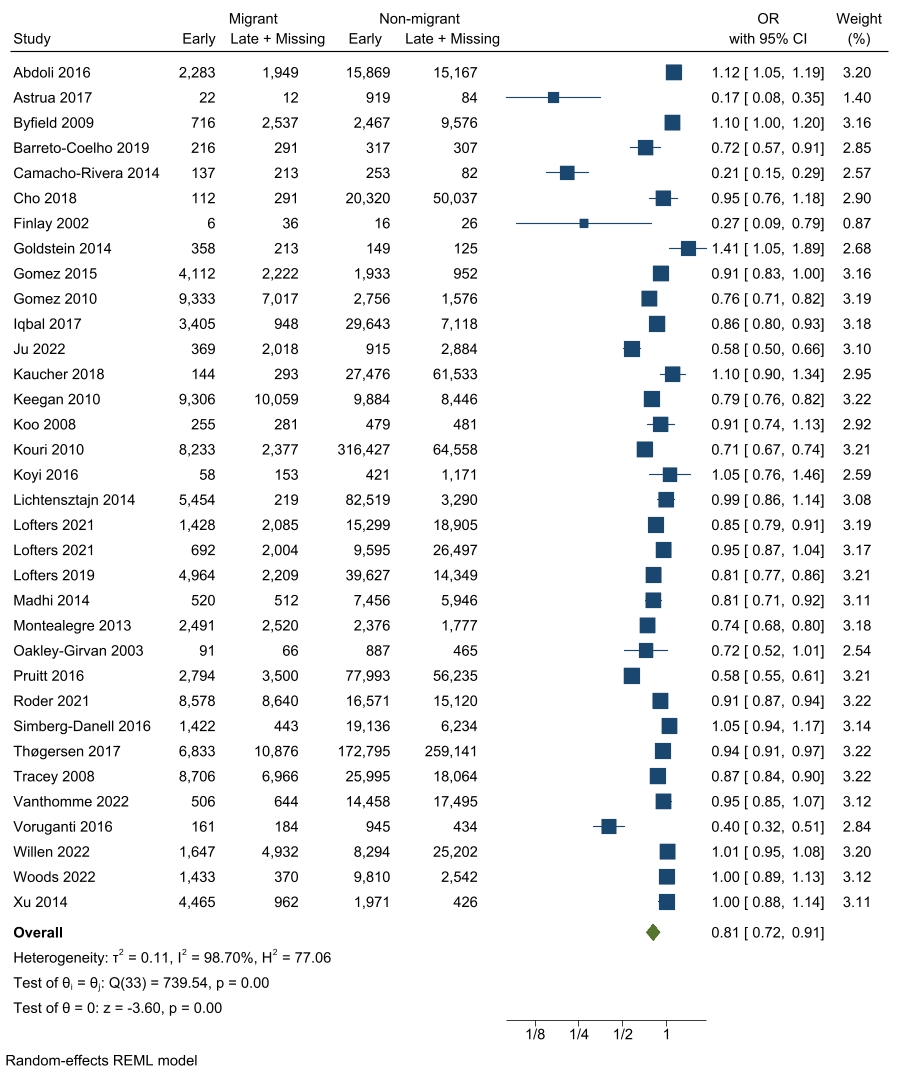


**Figure 10:** Forest plot of all studies pooled with missing data included as late stage, comparing early stage cancer at diagnosis between migrants to non-migrants.


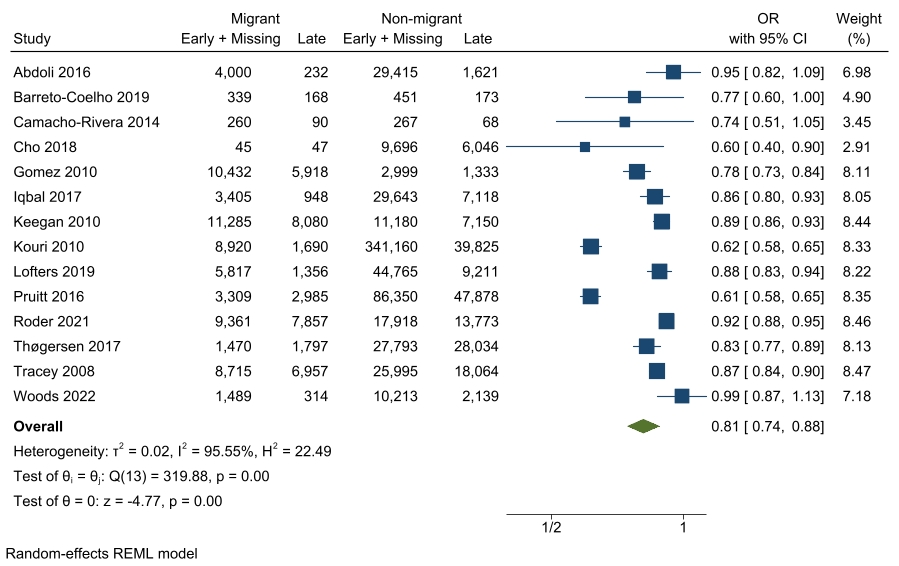


**Figure 11:** Forest plot of breast cancer studies pooled with missing data included as early stage, comparing early stage cancer at diagnosis between migrants to non-migrants.


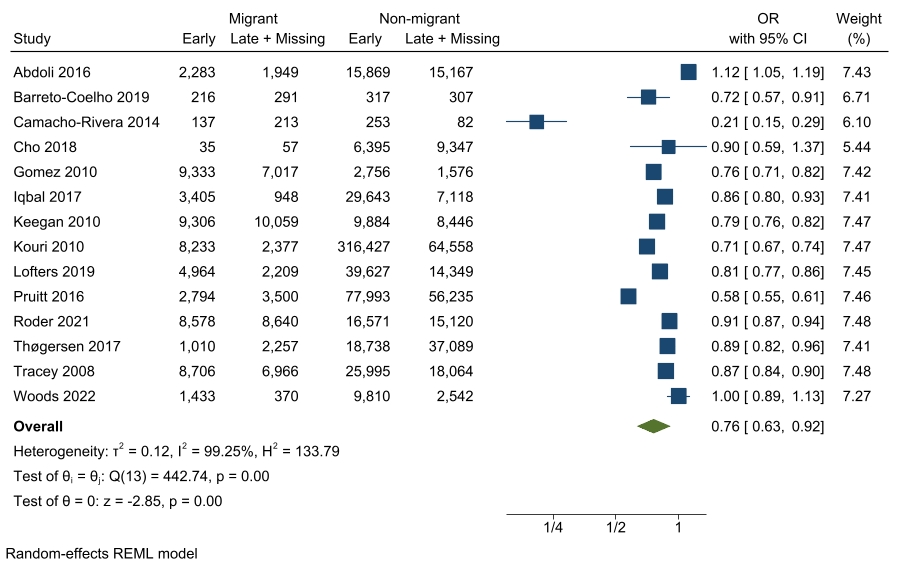


**Figure 12**: Forest plot of breast cancer studies pooled with missing data included as late stage, comparing early stage cancer at diagnosis between migrants to non-migrants.


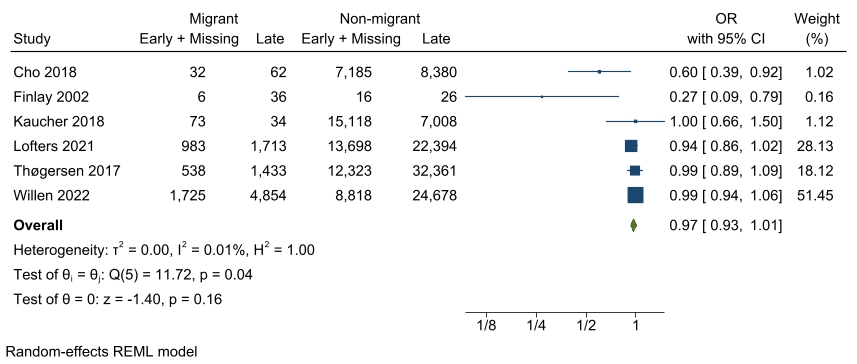


**Figure 13:** Forest plot of lung cancer studies pooled with missing data included as early stage, comparing early stage cancer at diagnosis between migrants to non-migrants.


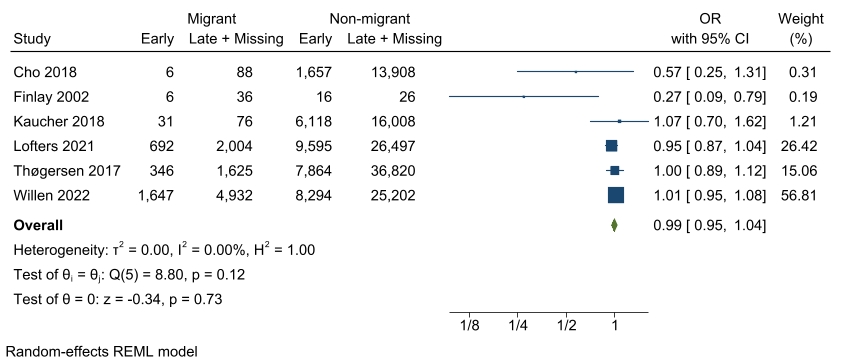


**Figure 14:** Forest plot of lung cancer studies pooled with missing data included as late stage, comparing early stage cancer at diagnosis between migrants to non-migrants.


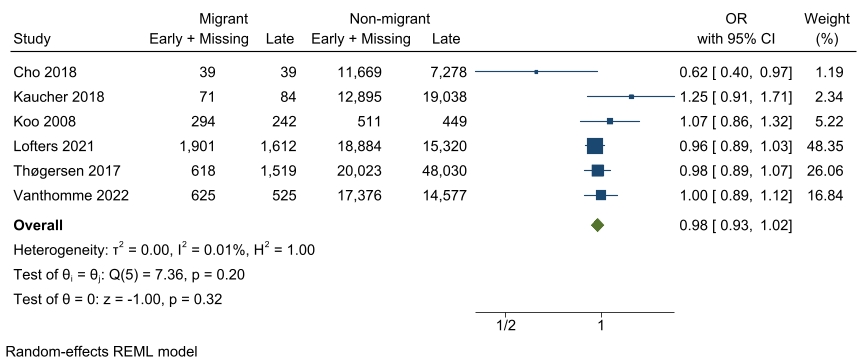


**Figure 15:** Forest plot of colorectal cancer studies pooled with missing data included as early stage, comparing early stage cancer at diagnosis between migrants to non-migrants.


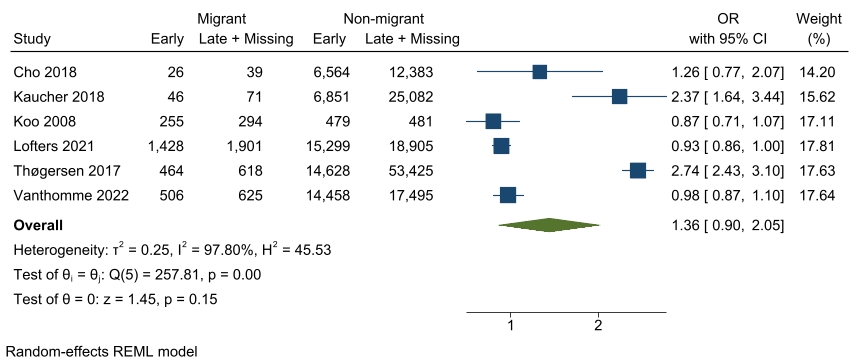


**Figure 16**: Forest plot of colorectal cancer studies pooled with missing data included as late stage, comparing early stage cancer at diagnosis between migrants to non-migrants.


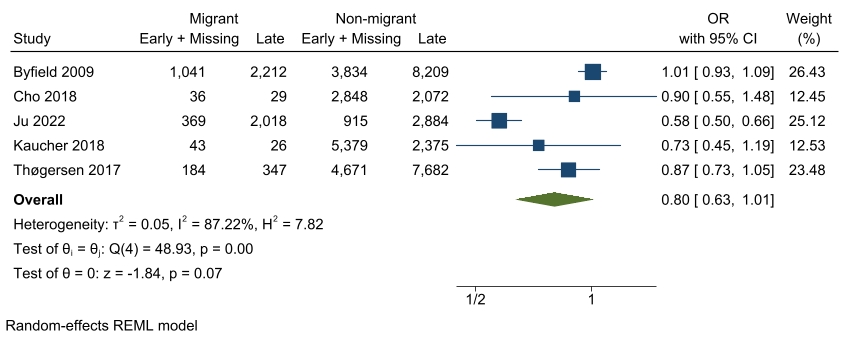


**Figure 17:** Forest plot of UGI cancer studies pooled with missing data included as early stage, comparing early stage cancer at diagnosis between migrants to non-migrants.


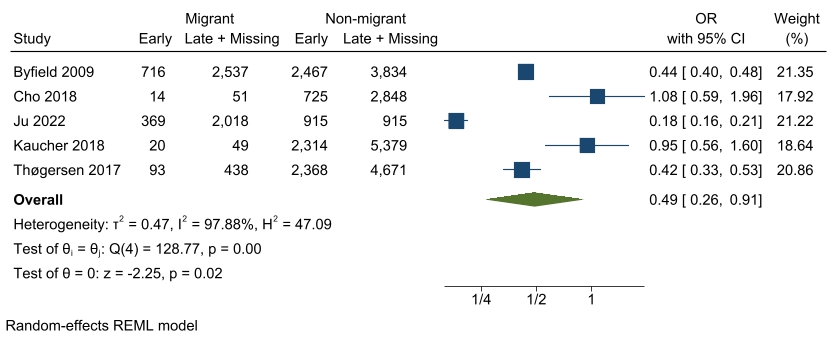


**Figure 18:** Forest plot of UGI cancer studies pooled with missing data included as late stage, comparing early stage cancer at diagnosis between migrants to non-migrants.


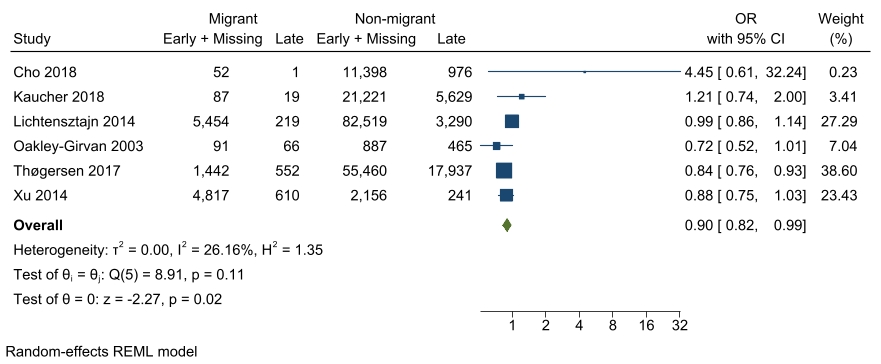


**Figure 19:** Forest plot of prostate cancer studies pooled with missing data included as early stage, comparing early stage cancer at diagnosis between migrants to non-migrants.


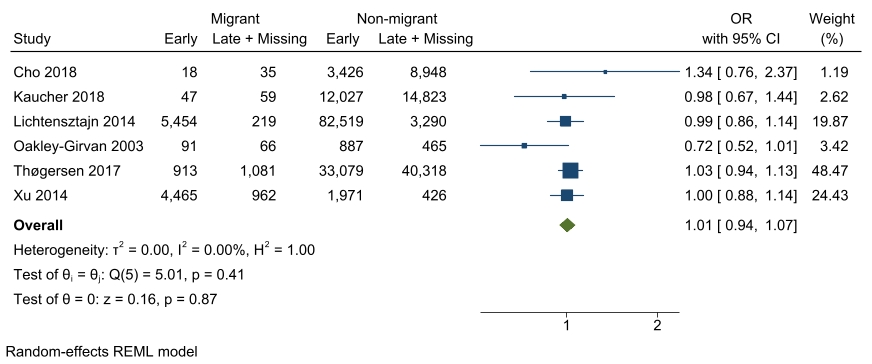


**Figure 20:** Forest plot of prostate cancer studies pooled with missing data included as late stage, comparing early stage cancer at diagnosis between migrants to non-migrants.


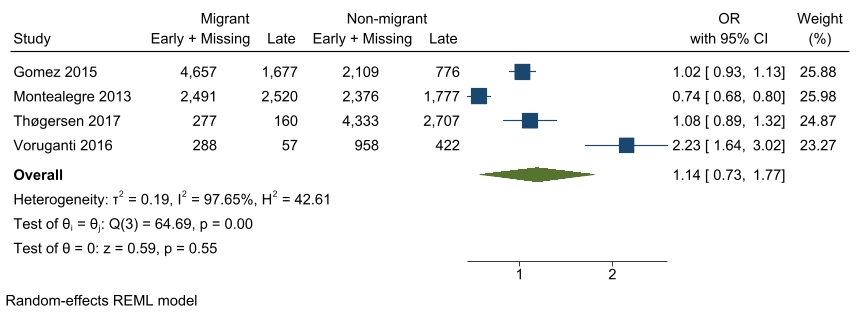


**Figure 21:** Forest plot of cervical cancer studies pooled with missing data included as early stage, comparing early stage cancer at diagnosis between migrants to non-migrants.


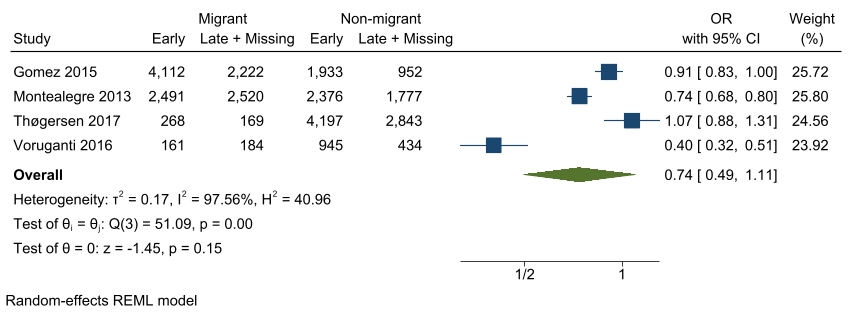


**Figure 22**: Forest plot of cervical cancer studies pooled with missing data included as late stage, comparing early stage cancer at diagnosis between migrants to non-migrants.

**Sensitivity analysis**

Two types of sensitivity analysis were performed. Firstly, an imputation analysis of missing data. Missing data was imputed as either early or late stage at diagnosis to whether it impacted the outcome.

Secondly, we instead of the early vs late stage at diagnosis classification, where appropriate, sensitivity analyses were conducted where stage at diagnosis data was dichotomised into “local” and “non-local”. Local stage at diagnosis was defined as either stage I or local depending on the cancer stage classification system. Non-local stage at diagnosis was defined as either stage II to IV or regional and distant.

| **Cancer type** | **Original analysis (OR [95% CI], I^2^)** | **Imputation analysis**  **(OR [95% CI], I^2^)** | **Local vs. non-local classification**  **(OR [95% CI], I^2^)** |
| --- | --- | --- | --- |
| **Overall** | 0.85 [0.78-0.91], 97% | Early: 0.87 [0.80-0.95], 97%  Late: 0.81 [0.72-0.91], 99% | 0.84 [0.77-0.91], 97% |
| **Breast** | 0.78 [0.70-0.87], 97% | Early: 0.81 [0.74-0.88], 96%  Late: 0.76 [0.63-0.92], 99% | 0.82 [0.74-0.91], 97% |
| **Lung** | 0.99 [0.94-1.03], 0% | Early: 0.97 [0.93-1.01], 0%  Late: 0.99 [0.95-1.04], 0% |  |
| **Colorectal** | 0.97 [0.88-1.05], 45% | Early: 0.98 [0.93-1.02], 0%  Late: 1.36 [0.90-2.05], 98% |  |
| **UGI** | 0.86 [0.64-1.16], 89% | Early: 0.80 [0.63-1.02], 87%  ^†^Late: 0.49 [0.26-0.91], 98% |  |
| **Prostate** | 0.92 [0.85-0.99], 0% | Early: 0.90 [0.82-0.99], 26%  ^††^Late: 1.01 [0.94-1.07], 0% |  |
| **Cervical** | 0.97 [0.78-1.21], 90% | Early: 1.14 [0.73-1.77], 98%  Late: 0.74 [0.49-1.11], 98% |  |
| **Melanoma** | 0.50 [0.16-1.52], 87% | Insufficient data for imputation analysis |  |
| † When missing data imputed as late stage at diagnosis migrants were found to be significantly less likely to be diagnosed with early stage UGI cancer.  †† When missing data imputed as late stage at diagnosis, stage at diagnosis for migrants was found to be not significantly different for prostate cancer. | | | |
